# Stem Cells-Derived Extracellular Vesicles: Potential Therapeutics for Wound Healing in Chronic Inflammatory Skin Diseases

**DOI:** 10.3390/ijms22063130

**Published:** 2021-03-19

**Authors:** Enzo Manchon, Nell Hirt, Jean-David Bouaziz, Nabila Jabrane-Ferrat, Reem Al-Daccak

**Affiliations:** 1 Institut National de la Santé et de la Recherche Médicale (Inserm) UMRS976, Université de Paris, Hôpital Saint-Louis, 75010 Paris, France; enzo.manchon@inserm.fr (E.M.); nell.hirt@inserm.fr (N.H.); jean-david.bouaziz@aphp.fr (J.-D.B.); 2 Department of Dermatology, AP-HP, Hôpital Saint-Louis, 75010 Paris, France; 3 Infinity, Université Toulouse, CNRS, Inserm, CEDEX 3, 31059 Toulouse, France; nabila.jabrane-ferrat@inserm.fr

**Keywords:** extracellular vesicles, exosomes, stem cells, skin, wound healing, chronic inflammation, regenerative medicine

## Abstract

Endosome-derived small extracellular vesicles (EVs), often referred to as exosomes, are produced by almost all, if not all, cell types, and are critical for intercellular communication. They are composed of a lipid bilayer associated with membrane proteins and contain a payload of lipids, proteins and regulatory RNAs that depends on the parental cell physiological condition. By transferring their “cargo”, exosomes can modulate the phenotype of neighboring and distant cells. Stem cells (SC) were widely studied for therapeutic applications regarding their regenerative/reparative potential as well as their immunomodulatory properties. Whether from autologous or allogeneic source, SC beneficial effects in terms of repair and regeneration are largely attributed to their paracrine signaling notably through secreted EVs. Subsequently, SC-derived EVs have been investigated for the treatment of various diseases, including inflammatory skin disorders, and are today fast-track cell-free tools for regenerative/reparative strategies. Yet, their clinical application is still facing considerable challenges, including production and isolation procedures, and optimal cell source. Within the emerging concept of “allogeneic-driven benefit” for SC-based therapies, the use of EVs from allogeneic sources becomes the pragmatic choice although a universal allogeneic cell source is still needed. As a unique temporary organ that ensures the mutual coexistence of two allogeneic organisms, mother and fetus, the human placenta offers a persuasive allogeneic stem cell source for development of therapeutic EVs. Advancing cell-free therapeutics nurtures great hope and provides new perspectives for the development of safe and effective treatment in regenerative/reparative medicine and beyond. We will outline the current state of the art in regard of EVs, summarize their therapeutic potential in the context of skin inflammatory disorders, and discuss their translational advantages and hurdles.

## 1. Introduction

The last three decades have witnessed a wealth of studies to provide the proof-of-concept in stem cells (SC)-based regenerative strategies to manage diseases that cannot be treated. Nonetheless, the development of clinically viable regenerative therapies remained challenging because SC faced major hurdle from immunological barriers. For instance, the nature and magnitude of host immune response to a given cell-based therapy are governed by several factors including cell properties and the degree of class I and II human leukocytes antigen (HLA) mismatch as well as by the mode of action and clinical efficacy [1,2]. To circumvent the barrier issues, autologous SC became an obvious choice for regenerative therapies. Yet, the demanding logistics to achieve immediate availability of sufficient numbers limited the potential use of autologous cells including iPSCs [3]. The well-documented ability of allogeneic adult SC to evade and/or regulate the host immune system comforted their consideration as a pragmatic choice that offers an efficient way for the development of off-the-shelf regenerative therapies with appropriate number of cells [4,5,6,7,8]. This paradigm shift in SC allogenicity led to the concept of “allogeneic-driven-benefit” [9]. As the field evolves it become evident that both autologous or allogeneic infused SC do not persist long enough to achieve direct regenerative/reparative effects. Most of the beneficial effects of transplanted cells are rather mediated through paracrine signaling pathways that promote endogenous tissue regeneration and repair. In this context, the immunomodulatory/anti-inflammatory properties of various types of SC are highlighted today as integral to their paracrine beneficial signaling in various disorders [9,10].

Extracellular vesicles (EVs) are natural highly conserved cell-derived vesicles secreted by various cell types including SC to ensure local and remote cell-to-cell communication. EVs carry lipids, small RNAs and proteins that can be transferred from parent cells to target cells and regulate their activities [11]. Small-sized EVs or exosomes are proposed today, as the main mediators of SC paracrine beneficial effects. The in vivo visualization of small-size EVs in tissues treated with SC, such as injured mouse heart, [12,13] further supported the role of EVs as important players mediating the wholesome effects of therapeutic SC [14].

This notion raised hope for the development of novel cell-free therapies that would overcome the hurdles of using SC in regenerative medicine. By interacting with different cells, including immune cells, EVs can provoke and maintain the regenerative/reparative processes. In regard of their remarkably broad biological functions and their capacity to transport large molecules, EVs offer a unique platform for the development of novel therapeutic strategies for a variety of diseases and disorders, including wound healing in chronic inflammatory skin diseases. In this review, we summarize the current knowledge about SC-derived EVs and their potential to promote immunomodulation and re-epithelialization in the context of chronic skin inflammation and wound healing. We also discuss challenges that might cause their loss in translation.

## 2. Extracellular Vesicles/Exosomes: General Aspects

**Figure 1:**
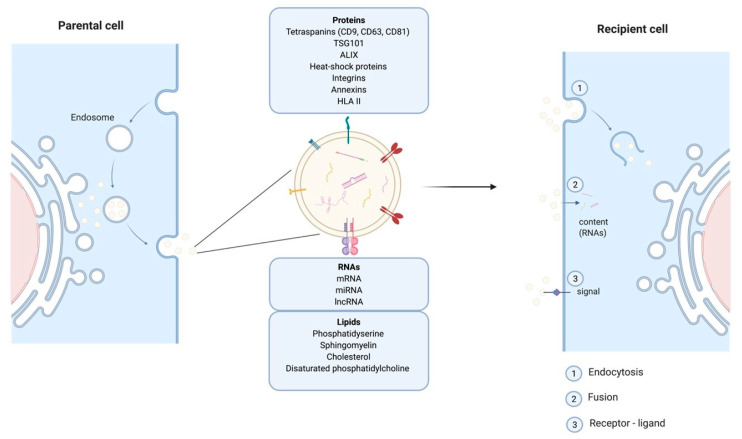
Schematic representation of exosomes endosomal budding detailing their different components and mode of action on recipient cells.

Cells release EVs of different sizes and intracellular origin which can be classified as ectosomes, exosomes, and apoptotic bodies [15]. Apoptotic bodies are a product of apoptosis and contain fragments from the dying cells, their size range from 50 to 5000 nm. Ectosomes (or microvesicles) result from protrusions of the plasma membrane that eventually detach and are shed in the extracellular space, their diameter is between 50 and 500 nm. The smallest EVs also called exosomes (Exs), category of interest referred to thereafter as EVs/Exs, have a diameter ranging between 50–150 nm. EVs/Exs arise from larger intracellular vesicles called multivesicular bodies (MVBs). They are secreted via exocytosis as a consequence of fusion between MVBs and the plasma membrane (Figure 1). Classical EVs/Exs express CD63, CD81, CD9 markers while the non-classical express CD63 and CD81 but lack CD9. EVs/Exs can also express several other proteins including heat-shock proteins (Hsp60, Hsp70, and Hsp90), programmed cell death 6 interacting protein (Alix/PDCD6IP), tumor susceptibility gene 101 (Tsg101), and clathrin h [16]. A wide range of cell types including SC, immune cells and cancer cells, produces EVs/Exs as mediators of their paracrine effects. EVs/Exs function is governed by the payload of lipids, proteins and different types of RNAs (mRNA, miRNA, lncRNA, etc.) originating from the parent cell [16].

EVs/Exs are considered as one of the major modes of cellular communication. They exert local and long-range action to impact other tissues. Their intercellular communication can be conferred by mediators expressed at their surface, or delivery of their “cargo” into target cell lumen after internalization through fusion or endocytosis [17]. Within these properties EVs/Exs are proposed as essential actors in tumorigenesis and distant metastasis development [18]. However, EVs/Exs are also key elements of SC-mediated paracrine regulation of cells/tissues repair and regeneration [14,19].

Given their growing importance in modern regenerative/reparative medicine, various techniques have been adopted to facilitate the challenging isolation of EVs/Exs. Ultracentrifugation, consecutive centrifugation, ultrafiltration, immunoaffinity precipitation or size exclusion chromatography, as well as high-resolution density gradient fractionation in combination with direct immunoaffinity capture have been used with continuous ameliorations to isolate various populations of EVs [20,21]. However, each approach has different pros and cons in regard of purity and number of isolated particles. For example, ultracentrifugation is efficient to remove several contaminants, but it is time consuming and not always suitable for EVs/Exs isolation from small clinical samples. Ultrafiltration is fast and results in highly pure vesicles, but the disadvantage comes from the difficulty to remove contaminating proteins which could be problematic for clinical purposes [20,21]. These examples illustrate the current challenges of improving isolation and purification technique for EVs/Exs. It is accepted that none of the up-to-date developed techniques can permit to clearly separate different types of EVs rendering pure EVs/Exs fractions extremely hard to obtain. Most preparations could be called “exosome-enriched fractions” of EVs, explaining our choice to use the term EVs/Exs in this review.

Much of the interest in EVs/Exs was triggered by their biological properties and their function especially the delivery of their “cargo” to neighboring and distant cells. The differences in purification strategies and the heterogeneity of EVs/Exs preparations may confound their proper characterization, which is essential for their biological properties. Therefore, a combination of different methodologies is often applied to best characterize these nanovesicles. Among the most common, colorimetric dosage for protein concentration, Tunable-Resistive Pulse Sensing (TRPS), Dynamic Light Scattering (DLS) and Nanoparticle Tracking Analysis (NTA) which can precisely measure particles concentration (number of particles/mL) and size distribution of EVs/EXs [22]. Atomic-Force Microscopy (AFM) or Transmission Electron Microscopy (TEM), are both for visualization and characterization of EVs structure, morphology, and size, while Western blotting, polymerase chain reaction (PCR), microarray, next-generation sequencing (NGS) and lipidomic approaches are used to determine the content of EVs. Additionally, surface markers are characterized through flow cytometry approaches [23].

## 3. Extracellular Vesicles/Exosomes: Immune Properties

### 3.1. Activation of Immune Response

Typically, specific immune response is triggered upon direct interaction of peptide-loaded class I and II major histocompatibility complex (MHC) on antigen-presenting cells (APCs) with CD8^+^ or CD4^+^ T cells, respectively. T cell receptor (TCR) recognition of these peptide loaded MHC complexes leads to the formation of the immune synapse (IS), a stable interaction between the T cell and the APC. The synaptic zone allows the transfer of information between the two cells by means of trogocytosis, tunneling nanotubes and, polarized secretion of soluble factors and EVs/Exs. As EVs/Exs are formed by reverse budding of the multivesicular body, functional proteins specifically associated with plasma membrane are exposed on their outer surface [24,25]. Accumulating evidence has demonstrated that EVs/Exs can be transferred at a distance and mediate antigen presentation [26]. EVs/Exs loaded with peptides from Epstein-Barr virus (EBV), cytomegalovirus and influenza virus could directly induce in vitro the secretion of IFNγ by human peripheral CD8^+^ T cells, but the magnitude of T cell activation is 10 times lower than direct stimulation with APCs [27]. Antigen presentation also occurs in an indirect manner after EVs/Exs internalization by APCs and degradation of their peptide/MHC complexes. For instance, HLA-DR4-positive EVs/Exs loaded with a serum albumin peptide were able to stimulate T cells following internalization by HLA-DR4-positive APCs [28]. This suggests that EVs/Exs are able to transfer either the preformed peptide–MHC II complex or the specific peptide to MHC II molecules on APCs promoting specific T cell activation without further antigen processing. EVs/Exs can also trigger an immune response by delivering native antigens to APCs. Tumor-derived EVs/Exs are internalized by APCs, processed and cross-presented to cytotoxic CD8^+^ T cells, and vaccination of mice with tumor-derived EVs/Exs induces a potent CD8^+^ T cell–mediated anti-tumor response against not only the parental tumor, but also other tumors expressing similar tumor antigens [29].

Triggering an immune response by EVs/Exs also occurs via mechanisms other than antigen presentation. EVs derived from bacteria-infected macrophages stimulate macrophages and neutrophils to secrete pro-inflammatory cytokines [30]. In diabetes, EVs/Exs-mediated transfer of specific miRNAs to pancreatic cells lead to T-cell death and expression of chemokine genes, which would in turn further the infiltration of activated T cells [31]. In cancer, miR-21 and miR-29a from tumor-derived EVs/Exs bind to Toll-like receptors, such as human TLR8, and lead to TLR-mediated NFκB activation and secretion of pro-metastatic inflammatory cytokines, which in turn promotes tumor growth and metastasis [32]. Overall, the capacity of some EVs/Exs to activate the immune system could be beneficial to diseases where immune response is defective but also in cancer to improve anti-tumor response.

### 3.2. Regulation/Suppression of Immune Response

EVs/Exs activate the immune response, yet, depending on their cellular origin, they can also act as key immunoregulators/suppressers. Human regulatory T cell-secreted EVs/Exs suppress effector T cell proliferation and cytokine production in vitro, and can prevent allograft rejection in vivo [33]. Tumor-derived EVs/Exs express FasL and TRAIL membranous death ligands as well as regulatory proteins such as PD-L1 and CD40L [34,35]. These surface molecules could trigger the apoptotic death of activated T cells, inhibit effector T cells activity and promote generation of regulatory T cells. Through their “cargo” of proteins, RNAs, and lipids, tumor-derived EVs/Exs down-regulate anti-tumor immune responses, and are currently recognized as key actors of tumor-induced immunomodulation/suppression. In addition to regulating anti-tumor T cells response, tumor-derived EVs/Exs can directly suppress NK cell anti-tumor response, and can facilitate angiogenesis and wound healing [36,37,38].

EVs/Exs secreted by adult stem cells, including mesenchymal stem cells (MSCs) and cardiac stem/progenitor cells (CPCs), confer immunomodulatory/anti-inflammatory regenerative/reparative effects in animal models of diseases and tissue injury. Notably, MSC-derived EVs/Exs were more efficient than their parental cells in reducing the percentage of effector CD8^+^ and CD4^+^ T cells while increasing regulatory T cells in inflammatory arthritis mouse models [39], whereas CPCs-derived EVs/Exs were as efficient as their parental cells in myocardial infarction experimental models [40,41]. Similar to tumor-derived EVs/Exs, SC-derived EVs/Exs can down-regulate immune functions via direct interaction of surface PD-L1, or CD40L with extracellular proteins on immune cells. For instance, EVs/Exs from fetal SC express PD-L1 and mediate T cell suppression by inhibiting the CD3-zeta and JAK3 pathway [42], which is critical for T cell proliferation in response to antigen receptor cross-linking. Together these findings strongly supported the active contribution of EVs/Exs to the immunoregulatory/anti-inflammatory properties of adult and fetal SCs.

Interestingly, inflammation, often marking degenerative disorders and tissue injury, seems to reinforce the immunomodulatory/suppressive capacity of SCs-derived EVs/Exs. MSC treated with IFNγ and TNFα produce EVs/Exs with higher immunosuppressive/anti-inflammatory capacity directing the differentiation of M1 macrophages (pro-inflammatory) into an M2 (anti-inflammatory) phenotype with IL-10 production [43]. Hypoxia can also enhance the immunomodulatory and angiogenic properties of MSC-derived EVs/Exs [44,45]. Similarly, under inflammatory conditions, CPCs-derived EVs/Exs protect monocytes from spontaneous death and fine-tune their phenotypes towards anti-inflammatory/immunoregulatory profile enhancing repair and healing of injured heart [14,41].

In brief, EVs/Exs are at the center of the most exciting and growing field of SC secretomics. EVs/Exs not only display various immune relevant proteins at their surface but also carry a “cargo” of growth factors, microRNAs, long non-coding RNAs with tremendous immune regulatory activities as well as beneficial regenerative properties. As such, EVs/Exs became promising therapeutic strategies for autoimmune and inflammatory diseases but also for many degenerative diseases [46]. Indeed, as therapeutics, they might be useful in overcoming current limitations of SC-based strategies including tumor formation and other hurdles that might be caused by SC transplantation. In this context, furthering the characterization and understanding of EVs/Exs derived from various types of SCs is essential not only to elect an optimal cell source but also to develop cell-free therapeutics for various degenerative diseases and devastating injuries.

## 4. SC-Derived EVs/Exs and Skin Wound Healing

**Figure 2:**
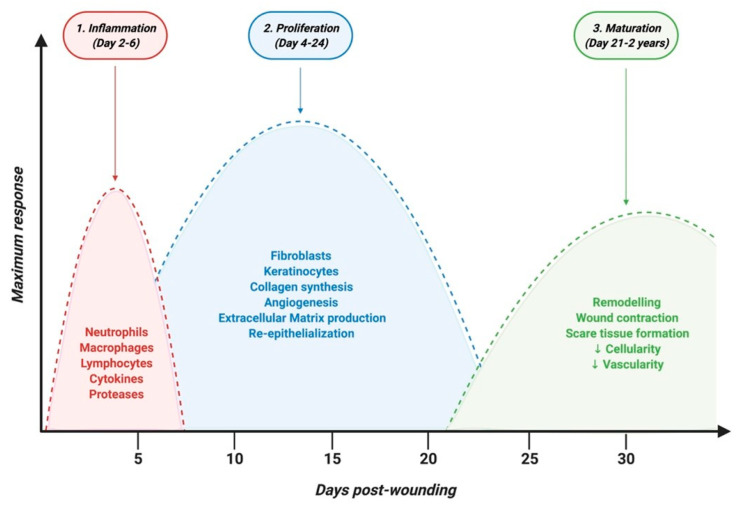
Timeline of physiological skin wound healing phases.

The skin, composed of epidermis, dermis, and hypodermis, represents 15% of the body, and is the first line of defense against external aggressions such as pathogens or UV light. Dysfunction or injury of the skin is highly prevalent and can lead to a range of diseases. It can result from trauma or surgical incision in the case of acute wounds but also from diseases such as chronic inflammatory or autoimmune skin diseases, which are marked by chronic skin wounds with serious healing defects. Keratinocytes and fibroblasts are main cellular composites of the skin and are critical for wound healing. Patients suffering from skin disorders not only face decreased quality of life, but also are often embarrassed about the appearance of their skin, which can cause an immense psychological burden. Skin wound healing is a complex process involving three major overlapping phases: inflammatory, proliferative and remodeling phases where several cell types are involved [47] (Figure 2).

### 4.1. Inflammatory Phase

The inflammatory phase is marked by the recruitment of inflammatory cells to the site of injury. Neutrophils are the first arrivals, within the first 24 h after injury, where they synthesize and secret various proteases and antimicrobial compounds to prevent infections [48,49]. Both neutrophils generated products and their apoptosis attract macrophages and lymphocytes, which will digest matrix remains and cellular debris, as well as potential microorganisms, to promote, over approximately 48 h, the cleaning of the injured zone. Macrophages will then start releasing several cytokines in order to restrain and resolve the inflammation and to activate tissue remodeling and regeneration. The first macrophages arriving to the wound exhibit the “classically activated” pro-inflammatory M1 phenotype, which are then finetuned by signals from the microenvironment to “alternatively activated” anti-inflammatory M2 phenotype [50]. If this physiological process of inflammation is abnormally sustained, it will lead to chronic inflammation and wounding that needs to be alleviated. This requires activation of M2 macrophages, reduction of NK and T cells subsets activation and proliferation, and promotion of regulatory T cells expansion.

**Figure 3:**
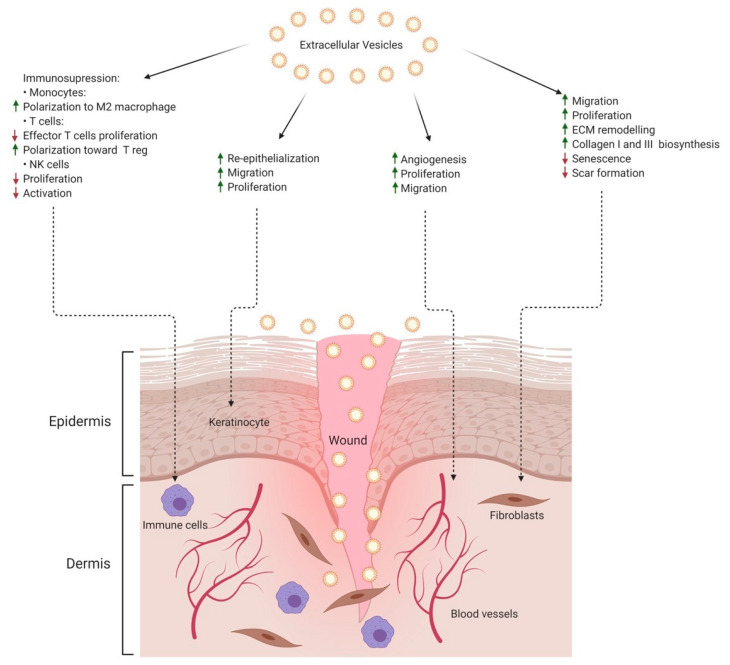
Potential effects of stem cell (SC)-derived EVs/Exs on skin wound healing.

Accumulating evidence suggest that SC-derived EVs/Exs have the potential to optimize all three phases of wound healing due to their capacity to control inflammation, stimulate cell migration and proliferation (Figure 3). In animal models of skin injury, bone marrow MSCs-derived EVs/Exs are able to (i) promote the switching of M1 macrophages towards the anti-inflammatory M2 phenotype, (ii) regulate the activation, differentiation, and proliferation of B cells and (iii) suppress effectors T cells as well as NK cells activation and proliferation [51,52,53]. It has become clear that EVs/Exs function can be affected by the microenvironment. For instance, pretreating MSC with LPS induces the production of EVs/Exs enriched with let7b, a miRNA that regulates the TLR4 pathway [54]. LPS-conditioned EVs/Exs inhibit the pro-inflammatory TLR4 pathway and activate anti-inflammatory STAT3/AKT serine/threonine kinase, resulting in the polarization of inflammatory macrophages towards an anti-inflammatory profile [54]. At the molecular level, the expression of miR-181c by MSC-derived EVs/Exs was reported as critical for promoting anti-inflammatory properties of macrophages through downregulating TLR-4 signaling pathway [55]. Similarly, preconditioning MSC under hypoxic conditions enriches their derived EVs/Exs with metabolites associated with anti-inflammatory activities, M2 macrophage polarization, and induction of regulatory T cells [45]. Strategies applying EVs/Exs from preconditioned MSC have been used in the treatment of chronic inflammation and wound healing, in vitro and in vivo, with promising results [54]. Knowledge about the direct suppressive effects of SC-derived EVs/Exs on T cells is still limited and calls upon further investigations. Nonetheless, in allogeneic skin-graft and graft-versus-host disease models, MSC-derived EVs/Exs regulate T cells activities via macrophages- or DCs-mediated pathways [56,57]. Thus, SC-derived EVs/Exs are prone to have anti-inflammatory properties, therefore, could be highly promising candidate for resolving skin chronic inflammation and wounding.

### 4.2. Proliferative Phase

Fibroblasts and keratinocytes are two main actors of the proliferative phase. They produce the extracellular matrix (ECM) and collagen and they govern wound contraction and re-epithelization [58]. Defective transition from the inflammatory to proliferative phase can cause persistent skin injury. Improving skin re-epithelialization is therefore critical to avoid persist and severe injuries.

EVs/Exs from various SC including hiPSC can modulate the functions of both fibroblasts and keratinocytes. In vitro and in vivo experimental models demonstrated that SC-derived EVs/Exs, actively contribute not only to collagen and elastin deposition and to wound healing, but also to migration and proliferation of dermal fibroblasts and keratinocytes [59,60,61,62]. These findings raised hopes for using EVs/Exs-based strategies to relief and treat inflammatory skin diseases with persistent injuries. Mechanistically, SC-derived EVs/Exs transfer various miRNA and non-coding RNA to fibroblasts and keratinocytes leading to the activation of several signaling pathways in these skin cells. These include wound healing pathways and regulatory factors, including cyclin-1, N-cadherin, PCNA, and collagens I, III, which together enhance migration and re-epithelialization [59,63,64,65,66]. In addition, induction of Wnt4-β-catenin signaling by EVs/EXs derived from human umbilical cord blood MSC is clearly involved in the beneficial effects on wound healing and skin repair [67]. Activation of both AKT and MAPK/Erk1/2 pathways by SC-derived EVs/Exs is also, respectively, involved in inhibiting stress-induced skin cell apoptosis, and in promoting their migration and proliferation [67,68,69].

Angiogenesis is another critical process occurring during the proliferative phase and is fundamental for the delivery of oxygen, nutrients, and growth factors to the damaged tissue [70,71]. iPSC-derived EVs/Exs promote angiogenesis and accelerate wound healing in experimental skin injury models through upregulation of several angiogenesis-related genes including VEGF-A, PDGF-A, EGF, and FGF-2 [68]. Of note, EVs/Exs from preconditioned SC show higher capacity to induce proliferation, migration, and angiogenesis promoting thus accelerated wound healing both in in vitro and in vivo experimental models [72,73].

### 4.3. Remodeling Phase

The remodeling phase, also called the maturation phase of wound healing, is associated with the switch from type III to type I collagen and complete closure of the wound. When collagen is laid down during the proliferative phase, it is disorganized and the wound is thick, showing the importance of this stage of wound healing in scarring. However, excessive scarring is the result of abnormal collagen production by myofibroblasts and excessive ECM production by fibroblast. Interestingly, SC-derived EVs/Exs have the potential to reduce scar formation. The efficiency of SCs-derived EVs/Exs in reducing scar formation at the end of wound healing have been suggested by several in vivo experimental models. These EVs/Exs are capable of favoring proper remodeling of ECM and scarring reduction by controlling collagen I and III production and deposition, matrix metalloproteinase-1 (MMP-1) expression, and inhibiting the transforming growth factor-B/SMAD2 pathway to promote the differentiation of fibroblasts into myofibroblasts [63,74]. miRNAs, like miR-21, -23a, -125b, and -145, within the EVs/Exs “cargo” have been implicated in reduction of scar formation and opened the perspective of using EVs/Exs enriched with such miRNAs to enhance the scarring process.

## 5. SC-Derived EVs/Exs as Nanomedicine Therapeutics for Chronic Skin Inflammation

Evs/Exs from various immune and non-immune cells contribute to the pathogenesis of various inflammatory skin diseases including psoriasis, atopic dermatitis (AD), as well as autoimmune disorders [75]. For instance, EVs/Exs from both keratinocytes and mast cells contribute to psoriasis inflammation through the activation of neutrophils and CD1a-reactive T cells, respectively [76,77]. In autoimmune inflammatory bullous pemphigoid, blister fluid-derived EVs/Exs containing a variety of inflammatory proteins contribute to the pathogenesis of this severe disorder [78]. Within these findings, EVs/Exs have been considered as potential biomarkers of inflammatory skin disorders but also regarded as ultimate therapeutic agents that can be even further engineered to deliver various drugs. Thus, the current state of knowledge on EVs/Exs accentuated the therapeutic potential of these SC-derived nanoparticles in the context of chronic skin inflammation and its associated chronic wounding, and encouraged the development of nanomedicine strategies to manage such disorders.

The ability of EVs/Exs to impact cells depends on their protein markers and cargo, which mimic the properties of their origin. Therefore, among the first considerations when developing an EVs/Exs-based therapeutic strategy is the cellular source and whether it is autologous or allogeneic, in order to avoid unwanted biological activity inherent to parent cells. For instance, MSC from healthy individuals or cancer patients may deliver a bioactive EVs/Exs cargo that inhibits or promotes tumor growth, respectively. Exosomal PD-L1 expression also changes during treatment with anti-PD-1 antibodies in melanoma as well as in head and neck cancers, and certain anti-cancer drugs can induce the release of exosomes that can effectively induce natural killer cell cytotoxicity. Whether anti-inflammatory drugs and monoclonal antibodies used as inflammatory skin diseases treatment regimen alter the bioactive cargo of EVs/Exs derived from patient cells remains an open question. Yet, within the above findings one may speculate similar scenarios. Thus, it would be preferable to use SC cells-derived EVs/Exs obtained from healthy allogeneic individuals, rather than patients’ autologous cells, to develop therapeutic strategies. In addition, besides being more pragmatic, the therapeutic potential of EVs/Exs from allogeneic sources has been proven higher or at least equal to that of EVs/Exs from autologous source in various in vitro and in vivo experimental model systems, including skin injuries [14,41,79,80]. The concept of “allogeneic-driven-benefit” is today fairly admitted, but, the optimal allogeneic cells, if any, are still under active research.

The human placenta as a unique temporary organ that ensures the mutual coexistence of the allogeneic organism of mother and fetus [81,82,83], is considered a natural model of human transplantation. Accordingly, it was suggested as a potential “universal source” for the development of allogeneic biotherapeutics. This notion is further facilitated by the non-invasive and fairly ethical accessibility of the organ and by the fact that placental EVs/Exs perform a myriad of functions from regulation of maternal immune reaction to the physiological development of the fetus [84,85,86]. Thus, placenta-derived EVs/Exs are likely endowed with intrinsic immunomodulatory regenerative/reparative capacity [87], and might be considered as “universal EVs/Exs” for the development of efficient nanomedicine strategies to manage chronic inflammatory disorders.

SC-derived EVs/Exs as biotherapeutics can be delivered through intravenous, subcutaneous, intraperitoneal, oral or even nasal administration. Regardless of the delivery route, EVs/Exs primarily accumulate in organs such as the liver, spleen, kidney, and lung [88,89]. Currently, information on SC-derived EVs/Exs biodistribution and retention time in skin wounds is still scarce. Nonetheless, because the retention time of EVs/Exs in different organs is in general short and depends on EVs/Exs origins [89,90], increasing EVs/Exs’ half-life as well as resistance to biodegradability through numerous engineering techniques is under active development. Among the recent advances, some in vivo studies showing that encapsulation of EVs/Exs in hydrogel extends their retention at injured sites and endows them with higher wound healing capacity [91,92].

Development of wound dressings containing SC-derived EVs/Exs is another innovative, non-invasive and simple way of delivering EVs/Exs to patients suffering from chronic skin inflammation. The composition of wound dressing can be also modulated to provide a proper microenvironment that would increase their benefits. In this regard, it has been shown in a rat model of chronic diabetic wounds that an EVs/Exs-based wound dressing composed of antioxidant polyurethane (PUAO) cryogel and supplemented with ADSCs-derived EVs/Exs, efficiently promotes angiogenesis, collagen remodeling, granulation tissue formation, and re-epithelialization, thus wound healing [93].

**Figure 4:**
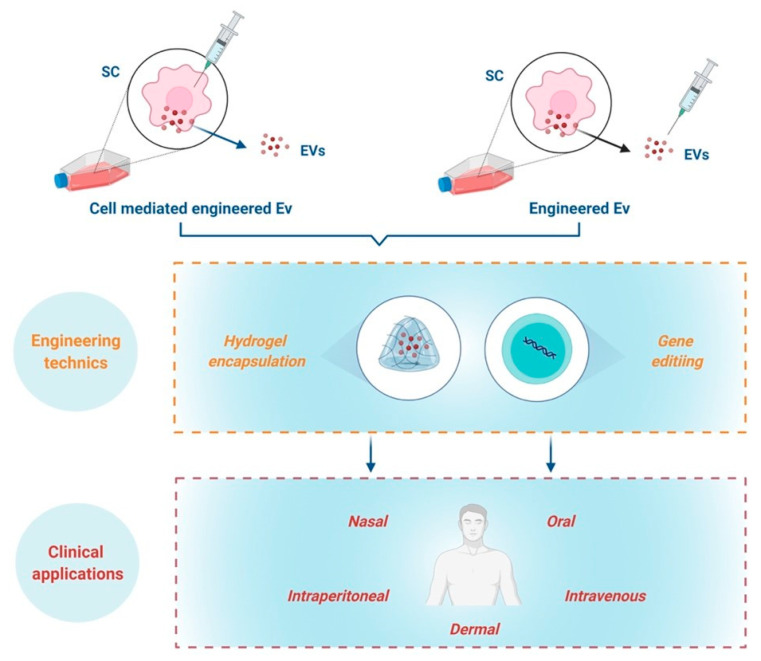
Schematic representation of engineering approaches towards clinical applications.

With the fast-track advances of gene editing techniques, such as CRISPR-Cas9, generating EVs/Exs tailored to enhance their immunomodulatory/anti-inflammatory regenerative/reparative capacities is today highly doable. Tailoring could be achieved either by engineering parent cells to overexpress molecule(s) or miRNA, which would result in production of EVs/Exs expressing these molecules, or directly engineering EVs/Exs with miRNA or molecules that enhance their benefits [88,94,95,96] (Figure 4).

The combination of gene editing technics to create modified EVs/Exs with addition of biomaterials such as hydrogel encapsulation and composite-loaded wound dressing are highly promising for management of chronic skin inflammation. However, these concepts are still at their infancy and call upon furthering investigations to reach the clinical translation of EVs/Exs-based nanomedicine to help patients suffering from chronic skin inflammation.

## 6. Current Limitations of EVs/Exs-Based Therapeutic Strategies

Cell-free EVs/Exs-based therapeutics permit to avoid some limitations of SC-based strategies including eventual rejection and short persistence while almost acting in a similar manner as whole cells. Despite considerable advances in the field, successful clinical translation of these promising therapeutic strategies is still facing various challenges.

The low productivity of pure EVs/EVs is among the first concerns because therapy requires large amounts. The poor yield after isolation, could indeed limit sufficient production for large-scale development for therapy. Some conditions like hypoxia or pH modifications can increase EVs/Exs yield, but such cells conditioning prior to EVs/Exs isolation could modify their “cargo” and decrease their purity. To what extent EVs/Exs should be pure depends on the experimental question and end usage. High purity is a must to attribute a function, but less purity and higher quantity are in general required for most therapeutic strategies where function is paramount not the definitive association of function with EVs/Exs. Several studies also indicated that the composition of the EVs/Exs “cargo” be it proteins or RNAs can be different and depends on different purification methods [97]. miRNA and potentially other molecules from the EVs/Exs “cargo” could cause undesirable effects, such as tumorigenesis, which may be caused by differences contaminating products. Thus, EVs/Exs collected with different methods, could differ in many aspects and properties, and there is no one-size-fits all procedure; mainly a balance between purity and quantity in function of EVs/Exs end-use to preserve particles properties and reduce the risk of adverse effects.

The eventual misallocation of EVs/Exs at sites other than the intended target upon administration is another current concern calling upon furthering the understanding of EVs/Exs bio-distribution to increase benefit and avoid undesired effects. The biology of miRNA is complex, and a given molecule could induce distinct and even opposite effects in different tissues. It means that administration of EVs/Exs therapy needs improvement to permit specific organs targeting.

## 7. Concluding Remarks & Future Directions

SC-derived EVs/Exs have proved their capacity to regulate the immune response towards promoting tissue repair in several conditions. Such properties are really promising, with great potential to translate to new efficient cell-free therapies for several pathologies. In the context of skin chronic inflammation, several recent publications provided sufficient proof-of-concept for SC-derived EVs/Exs as accelerator of wound healing process and resolvers of chronic inflammation. Today, we are at the stage of solving and optimizing various drawbacks of using EVs/Exs for therapies, including methods of isolation and characterization, large-scale production, optimization of cells culture conditions, and last but not least, protocols of administration. It is also critical to find the optimal cell source, since genetic modification tool are available. Immortalization of this optimal source is also required since senescence due to several culture passages affect the EVs/Exs “cargo”. This could allow the production of the optimal cell source for a specific pathology, which produce EVs/Exs with specific contents.

Regarding chronic skin inflammation and its associated defective wound healing, the current data show that similar beneficial effects could be obtained by EVs/Exs from different SCs. However, when comparing EVs/Exs from different SC types, the “cargo” is different. Different “cargo” would have different modulatory properties, meaning that “perfect EVs/Exs” do not yet exist. There are several skin disorders with no treatment available, using EVs/Exs for therapy could be a great solution to reduce pathological phenotypes. The idea is to find the optimal allogeneic SC to generate the best possible efficient EVs/Exs and to further tailor their fitness through gene editing to treat various inflammatory skin disorders.
